# Barriers to and delays in accessing breast cancer care among New Zealand women: disparities by ethnicity

**DOI:** 10.1186/s12913-015-1050-6

**Published:** 2015-09-18

**Authors:** Lis Ellison-Loschmann, Ridvan Firestone, Lucy Aquilina, Fiona McKenzie, Michelle Gray, Mona Jeffreys

**Affiliations:** Centre for Public Health Research, Massey University, P O Box 756, Wellington, 6140 New Zealand; University of Bristol, Tyndall Ave, Bristol, BS8 1TH UK; The International Agency for Research on Cancer, 150 cours Albert Thomas, Lyon, France; School of Social and Community Medicine, University of Bristol, Canynge Hall, 39 Whatley Rd, Bristol, BS6 7TQ UK

## Abstract

**Background:**

Unequal access to health care contributes to disparities in cancer outcomes. We examined the ethnic disparity in barriers to accessing primary and specialist health care experienced by New Zealand women with breast cancer.

**Methods:**

Women diagnosed with a primary invasive breast cancer between 2005 and 2007 were eligible. There were 1,799 respondents, *n* = 302 Māori (the indigenous population of NZ), *n* = 70 Pacific and *n* = 1,427 non-Māori/non-Pacific women. Participants completed a questionnaire listing 12 barriers grouped into three domains for analysis: personal; practical; and health care process factors, and reported the number of days between seeing a primary and a specialist care provider. Chi-squared, Fisher exact tests and logistic regression were used to assess uni- and multivariable differences in prevalence between ethnic groupings.

**Results:**

The prevalence of reporting three or more barriers was 18 % among Pacific, 10 % among Māori and 3 % among non-Māori/non-Pacific women (*P* <0.001). The most commonly reported barriers were fear (Māori women) and cost (Pacific and non-Māori/non-Pacific women). Ethnic differences in reported barriers were not explained by deprivation or diabetes prevalence. Women with diabetes reported a two-fold higher risk of experiencing barriers to care compared to those without diabetes (odds ratio [OR]: 2.06, 95%CI 1.20 to 3.57). Māori and Pacific women were more likely to face delays (median 14 days) in seeing a specialist than non-Māori/non-Pacific women (median 7 days); these differences were not explained by the reported barriers.

**Conclusions:**

Patterns of reported barriers to care differed according to ethnicity and were not explained by deprivation, or presence of co-morbidity. Māori and Pacific women are more likely to experience barriers to breast cancer care compared to non- Māori/non-Pacific women. We identified two key barriers affecting care for Māori and Pacific women; (a) delays in follow-up, and (b) the impact of co-morbid conditions. Future New Zealand work needs to focus attention on health care process factors and improving the interface between primary and secondary care to ensure quality health care is realised for *all* women with breast cancer.

## Background

Women in New Zealand (NZ) experience high breast cancer incidence and mortality rates compared to many other developed countries [1]. The burden of breast cancer is high and accounted for nearly 28 % of all cancer registrations and 16 % of all cancer deaths among NZ women in 2010 [2]. It is also worse among indigenous Māori women, who have a 60 % higher incidence of breast cancer [2] and a lower 4-year relative survival (86 % vs 92 %) [3] compared to non-Māori women. A growing body of literature indicates that access to care is a critical contributing factor to disparities in cancer outcomes [4, 5] although to date, little work has been done in NZ specifically in this area [5–7].

**Fig. 1:**
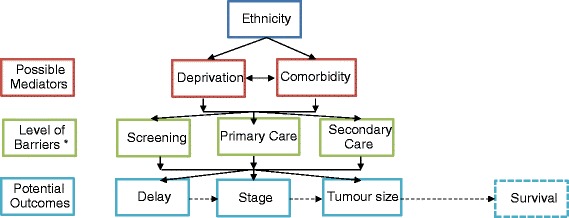
Conceptual model of access to cancer care. * Barriers: (1) Personal: embarrassment, not wanting to make a fuss, preferring not to know (fear), feeling it is pointless (2) Practical: cost, no transport, lack of childcare, can’t spare time/other priorities, pain (3) Health care process: not being able to get an appointment soon enough, not able to get in touch with the doctor, not trusting the health professional (Note: the dashed lines and box [Survival] are included for completeness, but have not been assessed in this analysis)

The recognised complexity involved in the provision of cancer services highlights the importance of access throughout the entire cancer care pathway with differences in health care access likely to be important mediators of cancer survival disparities [5, 8–11]. To guide our analysis, we developed a conceptual model (Fig. 1), in which we view ‘access’ as a multidimensional and multilevel process encompassing both “access to” and “access through” health care; the latter concept taking into account service quality. Access can be broadly considered across three interacting domains encompassing (a) health care processes; (b) patient factors; and (c) structural/health system factors [5, 9]. Examples of health care processes include availability of appointments, the effect of co-morbidity on treatment choices [10–12] or physicians’ perceptions and biases [10, 13, 14]. Patient factors relate to both practical issues that impact on decision-making such as family support and financial constraints [5, 9] as well as the influence of personal factors including fear or embarrassment [15–17]. Additionally, historical and contemporary structural health system factors including cultural safety across the health care spectrum, location and funding of services and the content of training programmes for health professionals are also important determinants of health care access and quality [5, 10, 11, 18–23].

Our aim was to understand ethnic differences in access to care among women with breast cancer in New Zealand. The specific hypotheses tested were: i) that the prevalence of barriers to primary and secondary care differ by ethnicity; ii) that some of these differences may be explained by deprivation and/or co-morbidity; iii) that barriers to care may explain some of the ethnic differences in delay in accessing care and iv) that screening services, and hence earlier diagnosis, are differentially used by women of different ethnicities.

## Methods

### Study population

The ‘New Zealand Breast Cancer Study’, a population-based case–control study, investigated risk factors throughout the lifecourse for breast cancer among three ethnic groupings: Māori, who make up approximately 15 % of the NZ population; Pacific (a composite group combining peoples self-identifying as Samoan, Cook Island Māori, Tongan, Tokelauan, Niuean, Fijian or from ‘other’ Pacific islands), who comprise 7 % of the population; European (74 %) (primarily from the United Kingdom and Europe) and Asian peoples (12 %) including those from South East Asia, Chinese and India [24, 25]. These last two groups are hereafter referred to as non-Māori/non-Pacific. Ethnicity data were collected based on the NZ census self-identified ethnicity and coded using a prioritized system whereby people who identify with more than one ethnic group are assigned to a single mutually exclusive category based on an established (Māori, Pacific, non-Māori/non-Pacific) hierarchy [26]. Thus, anyone identifying as Māori is coded as Māori regardless of any other ethnic group that person may have recorded; anyone identifying with a Pacific Island ethnic group was coded as Pacific unless they also identified as Māori and those coded as non-Māori/non-Pacific will not have identified as having either Māori or Pacific ethnicity.

The study design and methods have been published previously [27]. Eligibility was defined as having a primary invasive breast cancer registered on the NZ Cancer Registry (NZCR) between 1 April 2005 and 30 April 2006. Over-sampling of Māori and Pacific women involved inviting women diagnosed with breast cancer for a further 12 months (to 30th April 2007) to ensure sufficient statistical power for ethnic-specific analyses. The response rate was 78 % in non-Māori/non-Pacific women (*n* = 1,427), 46 % in Pacific women (*n* = 70), and 81 % in Māori women (*n* = 302). Written consent was obtained from all participants and ethical approval was granted by the Central Health and Disability Ethics Committee (WGT/03/12/126) of NZ. In this study, analyses are based solely on the cases.

### Data collection and assessment of barriers to care

Participants completed a detailed questionnaire socio-demographics, health behaviours, anthropometry, reproductive history, occupational history; and access to primary care and breast cancer treatment services [27] with most (89 %) questionnaires self-completed in English and returned by post. Other modalities for Māori and Pacific women included translation (<1 % of participants), telephone (8.6 %), or a face-to-face interview (1.8 %).

An area-based measure, the NZ Deprivation Index 2006 [28], assessed level of deprivation, using variables derived from the 2006 census, based on place of residence at the time of diagnosis, and was analysed in quintiles.

The list of barriers included in the questionnaire, focused on health care process and patient factors (divided into practical and personal factors), and was generated by the authors through a literature review, and tested in the pilot phase of the study [27]. A response option of “Other’ was also included where women could write in any additional barriers experienced which were not included on the list. To investigate the role of co-morbidity in determining barriers to care, we used a self-reported measure of doctor-diagnosed diabetes (*Have you ever been told by a doctor that you have diabetes or sugar in the blood’* – *Yes/No*). This measure was chosen, as diabetes prevalence in NZ is strongly patterned by ethnicity [29] and we had little data on additional co-morbidities.

To further understand the role of access to screening among women with breast cancer, we performed an analysis of tumour size and stage according to age. All women in NZ aged 45–69 years are eligible for free mammography through Breast Screen Aotearoa, the national screening programme [30]. We therefore categorized women according to eligibility for free mammography, and examined proportions of women diagnosed at an early stage, by ethnic grouping. Because of substantial missing data on stage at diagnosis, tumour size was also evaluated.

### Statistical methods

The potential barriers studied were tabulated individually. Chi-squared tests were used to test for differences in frequency, by ethnic grouping. In instances where one or more cells had fewer than five observations, exact tests were used in place of chi-squared tests. Although we purposely oversampled Māori and Pacific women, as all measures of prevalence were stratified by ethnicity, we did not need to account for sampling fractions in the analysis.

Given the sparse data, subsequent logistic regression was used to estimate odds ratios (OR) with 95 % confidence intervals (CI) for the association between ethnicity and each of the three barrier domain groupings, rather than for individual barriers. These OR were adjusted for age (continuous variable), then for age and deprivation quintile (categorical variable). The weeks and days between seeing a primary care provider and a specialist were converted to days. Non-parametric Kruskal Wallis tests were used to test for differences in median number of days’ delay between ethnic groupings, given the skewed nature of the data.

## Results

Demographic and clinical characteristics of participants are shown in Table 1. Māori and Pacific women were younger, came from more deprived areas, had larger / more advanced tumours and were more likely to have diabetes than non-Māori/non-Pacific women.

**Table 1:** Characteristics of women in New Zealand with breast cancer, by ethnicity

	Māori	Pacific	Non-Māori/non-Pacific
	N (%)	N (%)	N (%)
Total	302 (100)	70 (100)	1427 (100)
Age (years)			
20–35	10 (3.3)	4 (5.7)	25 (1.8)
36–50	97 (32.1)	37 (52.9)	377 (26.4)
51–65	137 (45.4)	19 (27.1)	589 (41.3)
>65	58 (19.2)	10 (14.3)	436 (30.6)
NZDep (quintiles)			
Q1 (least deprived)	16 (5.3)	5 (7.1)	282 (19.8)
Q2	31 (10.3)	5 (7.1)	265 (18.6)
Q3	52 (17.2)	9 (12.9)	333 (23.4)
Q4	73 (24.2)	21 (30.0)	325 (22.8)
Q5 (most deprived)	130 (43.1)	30 (42.9)	218 (15.3)
Extent			
Local	136 (52.5)	28 (52.8)	712 (56.7)
Regional	116 (44.8)	23 (43.4)	528 (42.0)
Distant	7 (2.7)	2 (3.8)	16 (1.3)
Tumour size (mm)			
<10 mm	34 (12.8)	9 (15.8)	308 (23.6)
11–19	69 (25.9)	16 (28.1)	416 (31.9)
20–29	105 (39.5)	13 (22.8)	325 (24.9)
30+	58 (21.8)	19 (33.3)	255 (19.6)
Diabetes			
Yes	58 (19.2)	12 (17.1)	115 (8.1)
No	244 (80.8)	58 (82.9)	1,309 (91.9)
Eligible for free screening^a^			
% never screened	5 %	5 %	12 %
% tumours < 10 mm (*p* <0.001)	14 %	20 %	26 %
% localized stage (*p* <0.001)	47 %	40 %	53 %

^a^women aged 45–69 years only

### Barriers to primary care

Barriers faced by women attending a primary care provider are shown in Table 2. Overall, non-Māori/non-Pacific women reported the fewest barriers, and among women reporting more than 3 barriers, the proportion was highest among the Pacific women (18 %), followed by the Māori women (10 %), and lowest among the non-Māori/non-Pacific women (3 %) (*P* <0.001). Among the Pacific women, the most commonly reported barriers were cost, inability to get a suitably timed appointment, fear, and not trusting a health professional. For Māori women, the most commonly reported barriers were fear, cost, and not wanting to make a fuss. For non-Māori/non-Pacific women, the most common barriers were cost and not wanting to make a fuss. ‘Other’ primarily included text which expanded on those barriers already listed. The most common additional comment made by women was in regard to delay in being followed up (Māori *n* = 3; Pacific *n* = 2; non-Māori/non-Pacific *n* = 12). Lack of cultural support was noted by one Māori participant.

**Table 2:** Distribution of reported barriers to primary care by ethnicity, among women in New Zealand with breast cancer

	Māori	Pacific	Non-Māori/non-Pacific	*P*-value
Cost	15 (5 %)	8 (11 %)	41 (3 %)	<0.001
Couldn’t get an appointment soon enough or at a suitable time	10 (3 %)	7 (10 %)	21 (1 %)	<0.001
Couldn’t spare the time / other priorities	8 (3 %)	1 (1 %)	29 (2 %)	0.79
Didn’t want to make a fuss	13 (4 %)	3 (4 %)	38 (3 %)	0.19
Had no transport to get there	4 (1 %)	3 (4 %)	9 (<1 %)	0.012
Couldn’t get in touch with the doctor or other professional	3 (1 %)	1 (1 %)	6 (<1 %)	0.12
Lack of childcare	2 (<1 %)	3 (4 %)	9 (<1 %)	0.023
Embarrassment	6 (2 %)	3 (4 %)	14 (1 %)	0.031
Pain or discomfort	7 (2 %)	3 (4 %)	8 (<1 %)	0.001
Prefer not to know condition / fear of being unwell	21 (7 %)	7 (10 %)	29 (2 %)	<0.001
Do not trust health professional	9 (3 %)	6 (9 %)	9 (<1 %)	<0.001
Feel that it is pointless	3 (1 %)	3 (4 %)	4 (<1 %)	0.002
Other	18 (6 %)	7 (10 %)	36 (3 %)	<0.001

The table shows the number of women who reported each item as a barrier; totals therefore sum to larger than the number of participants, as each woman could record more than one. *P* values derive from chi-squared tests, comparing proportions across the three ethnic groupings. In instances where there were five or fewer women in a particular cell, the *P* value reported is from a Fisher exact test

### Barriers to cancer specialist care

Barriers faced by women in seeing a specialist are shown in Table 3. Among Pacific women, 13 % reported facing three or more barriers, compared to 7 % among Māori women and 3 % non-Māori/non-Pacific women (*P* <0.001). Compared to barriers reported for the primary care provider, for specialist care, cost and inability to get a timely appointment were reported more frequently by all ethnic groupings. For Māori and non-Māori/non-Pacific women, the remainder of the barriers were reported at a similar frequency to the primary care provider analysis, whereas for Pacific women, these were reported less frequently. Specific issues reported more often by Māori compared to other women were not being able to spare the time and not having transport. Both Māori and Pacific women reported preferring not to know / fear of being unwell and concerns about pain as barriers to seeking care.

**Table 3:** Distribution of reported barriers to seeing a cancer specialist, by ethnicity, among women in New Zealand with breast cancer

	Māori	Pacific	Non-Māori/non-Pacific	*P*-value
Cost	21 (7 %)	12 (17 %)	75 (5 %)	<0.001
Couldn’t get an appointment soon enough or at a suitable time	21 (7 %)	10 (14 %)	91 (7 %)	0.037
Couldn’t spare the time / other priorities	5 (2 %)	1 (1 %)	4 (<1 %)	0.012
Didn’t want to make a fuss	8 (3 %)	1 (1 %)	17 (1 %)	0.16
Had no transport to get there	8 (3 %)	1 (1 %)	4 (<1 %)	<0.001
Couldn’t get in touch with the doctor or other professional	4 (1 %)	2 (3 %)	12 (1 %)	0.16
Lack of childcare	2 (1 %)	1 (1 %)	7 (1 %)	0.29
Embarrassment	6 (2 %)	1 (1 %)	9 (1 %)	0.056
Pain or discomfort	6 (2 %)	2 (3 %)	3 (<1 %)	<0.001
Prefer not to know condition / fear of being unwell	14 (5 %)	4 (6 %)	16 (1 %)	<0.001
Do not trust health professional	7 (2 %)	4 (6 %)	7 (1 %)	<0.001
Feel that it is pointless	3 (1 %)	1 (1 %)	3 (<1 %)	0.045
Other	13 (4 %)	2 (3 %)	56 (4 %)	0.88

The table shows the number of women who reported each item as a barrier; totals therefore sum to larger than the number of participants, as each woman could record more than one. P values derive from chi-squared tests, comparing proportions across the three ethnic groupings. In instances where there were five or fewer women in a particular cell, the P value reported is from a Fisher exact test

### Co-morbidity and access to care

The prevalence of diabetes was 8 % in non-Māori/non-Pacific women, 19 % in Māori and 17 % in Pacific women (*P* <0.001). Having diabetes was not related to reported barriers across any of the three domain groupings, for either a GP or specialist, except for practical barriers in seeing a GP. Women with diabetes reported an over two-fold higher risk of facing such barriers compared to those without diabetes (age- and ethnicity-adjusted OR: 2.06, 95%CI 1.20 to 3.57). When stratified by ethnicity, it did not appear that the effect of having a co-morbidity on reported barriers to care differed by ethnic grouping, P (interaction) = 0.18.

### Ethnic differences in accessing cancer care

Table 4 shows the multivariable analysis of the associations between ethnicity and the three barrier domains. Māori and Pacific women were more likely than non-Māori/non-Pacific women to report barriers in all three domains, with the strongest associations being for Pacific women facing health care process barriers in seeing a GP. None of the associations were explained by deprivation or by having a co-morbidity.

**Table 4:** Multivariable associations between ethnicity and barriers to accessing care, among women in New Zealand with breast cancer

	Model 1	Model 2	Model 3
Māori women			
Primary care			
Personal barriers	2.06 (1.28 to 3.29)	2.06 (1.25 to 3.41)	2.15 (1.33 to 3.46)
Practical barriers	1.56 (0.99 to 2.47)	1.39 (0.85 to 2.26)	1.46 (0.92 to 2.33)
Health care process barriers	2.67 (1.48 to 4.79)	2.35 (1.25 to 4.41)	2.57 (1.41 to 4.66)
Specialist care			
Personal barriers	2.97 (1.68 to 5.22)	2.57 (1.40 to 4.71)	2.91 (1.63 to 5.18)
Practical barriers	1.64 (1.06 to 2.51)	1.60 (1.01 to 2.52)	1.60 (1.04 to 2.47)
Health care process barriers	1.17 (0.76 to 1.84)	1.10 (0.69 to 1.76)	1.15 (0.73 to 1.81)
Pacific women			
Primary care			
Personal barriers	2.28 (1.03 to 5.05)	2.49 (1.09 to 5.70)	2.30 (1.03 to 5.13)
Practical barriers	2.42 (1.20 to 4.87)	2.12 (1.02 to 4.40)	2.19 (1.07 to 4.46)
Health care process barriers	7.59 (3.65 to 15.81)	6.49 (2.98 to 14.15)	7.41 (3.52 to 15.58)
Specialist care			
Personal barriers	3.33 (1.32 to 8.40)	3.57 (1.36 to 9.42)	3.37 (1.33 to 8.58)
Practical barriers	2.75 (1.44 to 5.24)	2.78 (1.42 to 5.47)	2.68 (1.39 to 5.14)
Health care process barriers	2.26 (1.18 to 4.34)	2.16 (1.10 to 4.25)	2.29 (1.19 to 4.41)

Model 1: age adjusted; Model 2: age and deprivation adjusted; Model 3: age and co-morbidity adjusted

The reference group is non-Māori/non-Pacific, so all OR compare Māori or Pacific women to the non-Māori/non-Pacific grouping

### Delay in access to care

The median delay between seeing a primary and specialist care provider was 14 days (inter-quartile range [IQR] -7 to 18) for Māori women, 14 days (IQR −7 to 21) for Pacific women and 7 days (IQR −3 to 14) for non-Māori/non-Pacific women, *P* <0.001. Thus, at 14 days after seeing a primary care provider, three-quarters of non-Māori/non-Pacific women had seen a specialist, compared with half of Māori and Pacific women. Age-adjusted analyses show that Māori and Pacific women are more likely to wait over 7 days between seeing a primary and secondary care provider than non-Māori/non-Pacific women (Table 5). However, these ethnic differences in delays were not explained by the barriers which Māori and Pacific women reported facing when seeing a specialist.

**Table 5:** Multivariable associations between ethnicity and delay of over one week in seeing a specialist, among women in New Zealand with breast cancer

	Māori	Pacific
Specialist care		
Age-adjusted	1.30 (1.00 to 1.69)	1.57 (0.91 to 2.73)
+Personal barriers	1.30 (1.00 to 1.68)	1.57 (0.90 to 2.72)
+Practical barriers	1.29 (0.99 to 1.67)	1.52 (0.87 to 2.64)
+Health care process barriers	1.27 (0.98 to 1.66)	1.42 (0.81 to 2.50)
+All barriers	1.28 (0.98 to 1.67)	1.45 (0.82 to 2.55)

The reference group is non-Māori/non-Pacific, so all OR compare Māori or Pacific women to the non-Māori/non-Pacific grouping

### Access to screening services

We explored the relationship between ethnicity and extent of disease at diagnosis, among women in the 45–69 year age range who were eligible for mammographic screening. Five percent of Māori and non-Māori/non-Pacific women and 12 % of Pacific women reported never having been screened. Tumour size differed between ethnic grouping, with 14 % of Māori, 20 % of Pacific and 26 % of non-Māori/non-Pacific women having a tumour of under 10 mm (*P* <0.001). The proportion of cancers which were diagnosed at a localised stage were 53 % for non-Māori/non-Pacific women, 47 % for Māori and 40 % for Pacific women (*P* <0.001). In addition, staging data were missing for a further 33 % of Pacific women, presumably because the cancer had spread so far that surgery was not indicated. There was no association between stage of disease or tumour size and diabetes in any ethnic grouping.

## Discussion

To our knowledge, this is the largest study of barriers to care among women with breast cancer in NZ. Key findings were that Māori and Pacific women were more likely to report multiple barriers to accessing primary care compared to non-Māori/non-Pacific women, and that these differences were not explained by deprivation. The longer delays for Māori and Pacific women for seeing a specialist were likewise not explained by any of the reported barriers to seeing a specialist. Additionally, although there was no ethnic difference in the greater barriers experienced by women with versus without diabetes, the prevalence of diabetes was highest among the Māori and Pacific women. The implication is that determinants of barriers to care by ethnicity involve more than deprivation and are affected by co-morbidity.

The study had three main limitations. Firstly, all the data were self-reported. For some measures of barriers, this is appropriate, but we would like to have had other measures relating to, for example, screening. The reported levels of ‘never screened’ in the current study were very low compared with those reported by the NZ breast screening programme, for example, biennial screening rates for women in 2009–2010 for the 50–69 years age group was 70.1 % with significantly lower participation evident for the eligible Māori and Pacific populations (58.1 and 64.3 % respectively) [31]. We are therefore unsure of the validity of our figures for self-reported screening participation; it may be that information given to women about the reason for mammography is not sufficiently clear and that the distinction between screening and symptomatic presentation, which is often investigated with mammography, needs to be more fully explained.

A second limitation was that much of the data was collected several months after diagnosis. This was due to the ethics committee request that we not contact participants within eight months of being diagnosed, to minimise distress. Thus, the reported experiences of women could have changed in response to the disease outcome and treatment in the months following diagnosis. We also acknowledge that although we had a free text option there may have been other barriers experienced by women that were not covered by our questionnaire, for example, relating to experiences of ethnic discrimination.

Thirdly, the response rate for Pacific women was low. There were small numbers in some categories which affected the precision of the percentages and effect estimates; we used appropriate statistical methods for small sample sizes. Given the paucity of research conducted in this population group however, we think this study makes an important contribution in terms of increasing the limited evidence available regarding the experience of breast cancer in Pacific women.

Our findings are consistent with other literature on ethnic barriers to care in NZ. Income, education, housing and employment are well recognized as key determinants of health [5, 10, 18, 31]. The differential distribution of deprivation, living standards, occupation and employment status by ethnicity in NZ have ongoing consequences in terms of access to care and health outcomes [29, 32]. For example, the 2011/2012 NZ Health Survey of over 17,000 adults found that approximately 14 % had deferred visiting a GP in the previous year because of cost, with Māori, Pacific and low income groups experiencing the greatest levels of unmet need in the primary care area compared to other population groups [29]. These are much higher proportions than identified in the current study however, possibly because the survey data included mostly healthy participants whereas the majority of women with symptomatic breast cancer probably realised that they had a potentially serious health problem. In terms of personal barriers, Pacific and Māori women were more likely than their non-Māori/non-Pacific counterparts to report fear as a barrier to seeking care in the current study. High anxiety levels have been documented amongst women attending routine mammography screening, with Māori and Pacific women more likely to report feeling ‘very worried’ about breast cancer compared to NZ European and Asian women [17].

Tumour size and stage may be considered as proxy measures of health care access since these factors could, in many instances, be mitigated by earlier presentation and diagnosis.

We found ethnic differences in delays for referral to specialist care follow-up which have been reported in previous work [31, 33] along with a greater likelihood of larger tumours and later stage among Māori and Pacific compared to non-Māori/non-Pacific women which has also been documented [3]. With regard to comorbidity, even though the ethnic disparities in prevalence were as expected, for each ethnic grouping, the actual prevalence rate was higher than recently reported figures [29]. It can be argued that co-morbidity should improve the likelihood of early cancer detection, in that those with a co-morbid condition have more frequent and/or regular engagement with health services than those without co-morbidity [34]. However, in our study, diabetes was not associated with either tumour stage or size, suggesting that engagement with the health care system for a comorbid condition does not automatically lead to better cancer health care.

Our results point to a need for new research to determine what factors cause – and also can reduce – ethnic disparities in access to appropriate care, both apart from and in conjunction with, deprivation and comorbidity. For example, a few NZ studies have identified integration of primary and secondary care and enhancement of mainstream services as being important enablers for successfully negotiating the complex cancer services environment [6, 7]. Campbell *et al.* [8] note that nearly all the priorities for cancer services are affected by actions in primary care – reducing the risk of cancer, early detection, faster access to specialist treatment and, planning to ensure adequate follow-up support post treatment. The NZ Ministry of Health is currently investigating ways to improve the quality, timeliness and options for different treatment service models for patients along the whole cancer pathway [35, 36]. Other avenues of intervention might be to expand support and resources to ensure the proactive engagement of health providers with the communities they serve, since evidence indicates this type of approach has enabled NZ screening programmes to successfully increase participation rates [37, 38]. Although we were unable to examine the explicit role of structural factors in these analyses, their interaction and influence on health care process and individual level factors, as critical determinants of access to care, are well documented [5, 9–12, 18–20, 39–42]. Factors such as health provider biases and institutional racism have been found to be important determinants of access to care in NZ and overseas [10, 13, 14, 20] both with regard to the potential impact on stage at which patients present with their symptoms as well as issues of trust in health professionals as was highlighted particularly for Pacific women in this study. Other factors including centralisation of services, while practical, may also exacerbate inequalities in access where, for example, remoteness to treatment centres [22] are likely to have a differential impact on population groups with fewer resources to offset the often significant economic [40] and social costs [41, 42] of cancer care.

## Conclusions

We have identified ethnic differences in the prevalence and patterns of barriers to health care services that NZ women face. Māori and Pacific women more likely to experience barriers to breast cancer care compared to non- Māori/non-Pacific women with two key barriers affecting care identified: (a) delays in follow-up, and (b) the impact of co-morbid conditions. From these data, it is premature to make direct recommendations for practice. However, health care process factors relating to the interface between primary and secondary care services may be important considerations in terms of both delays in follow-up for Māori and Pacific women and the impact of co-morbid conditions on health care provision and outcomes. Future NZ work needs to focus attention on health care process factors and improving the interface between primary and secondary care to ensure quality health care is realised for *all* women with breast cancer.
